# Letter from B. P. Reese

**Published:** 1874-03

**Authors:** B. P. Reese

**Affiliations:** Stanton, Va.


STANTON, VA. December 19, 1874.

*Messrs. Editors:*—Why is it that so few in the profession are contributors to our Medical Journals? We find men of long experience, who have vast stores of useful information acquired by careful observation rarely or never contribute to our journals. In every town and county there are men who have devoted fifteen and twenty years of their lives actively engaged in the profession, unknown outside of the immediate vicinity, and whose experience goes down to the grave with them. We live only long enough to learn by experience to be useful, and when we arrive at that age with a retrospect of the past, we only behold the opportunities for success and distinction passed, beyond the hope only of what may be accomplished within a few years. I presume there is no man beyond the meridian of life who cannot look back and see where he has failed to use advantages which would have lead him to success. If this be true, it behooves us, as a duty to those who succeed us, to make known the information gained by practical demonstration of what we have learned to be true.

Practical experience such as we can carry to the bed-side of our patients is what we want and need, and whoever can give us this is always acceptable. Our journals are too often filled with speculative theories of fancied imagination or with articles eminating from the fertile brain of some book-worm, more to gratify the cravings of a desire for reputation than practical knowledge. The busy practitioner has not the time nor disposition to read such, but seeks where he may find something tangible and practicable to meet his daily wants. About two-thirds of the published articles are written by men of little experience, and often that experience would tell a *sad* tale upon their success. We are too prone to publish our success, and seek to gain eclat from perhaps undeserved merit, when often our failures would be of paramount advantage to those traveling the hidden path to eminence and distinction. I do not believe this reticence of our professional men is actuated from selfish motives or an unwillingness to let it be known what they have found from practical experience to be useful. They freely consult and advise when occasion arises, to give all the information they have bearing upon any particular case, and yet their reluctance to writing, prohibits them from giving it to the profession through the journals. In this brief, hasty communication, I have a suggestion to make, which, if perhaps acted upon, may prove advantageous to journalists, and certainly beneficial to the physician. Let the editor have his agent or agents to visit different members of the profession in city and county, and prevail upon them to give a history of some interesting cases which have come under their observation, and this agent write out in detail for the journal, and get the indorsement of the physician to its accuracy. I send you this for what it is worth, and will try and practice what I preach, by sending you some of my practical experience in cases which have come under my observation.

Very respectfully, B. P. Reese.

The above should command the earnest attention of the profession. The editors are powerless to accomplish the ends sought by the author, but our friends in every State and county should attempt the collection of all facts in the hands of their brethren for publication, for the common good. A hint we trust will be sufficient to put them to work.

